# Endovascular management of an unsual case of spontaneous Retroperitoneal Haemorrhage due to Fibromuscular Dysplasia

**DOI:** 10.1186/s42155-020-00157-8

**Published:** 2020-09-09

**Authors:** Juan David Molina-Nuevo, Lorena López-Martínez, María José Pedrosa-Jiménez, Enrique Juliá-Molla

**Affiliations:** Complejo Hospitalario Universitario de Albacete, Albacete, Spain

**Keywords:** Fibromuscular dysplasia, Retroperitoneal haematoma, Embolization, Angioplasty

## Abstract

**Background:**

Fibromuscular dysplasia (FMD) is an uncommon vascular disease that results in stenosis, dissection or aneurysmal degeneration. However, it can sometimes manifest atypically, as we show in this case.

**Case presentation:**

A 24-year old patient with no relevant medical history with severe left hypochondrium pain. The physical examination showed blood pressure levels of 160/90 mmHg. An abdominopelvic CT evidenced left retroperitoneal haematoma associated with active bleeding and left renal artery stenosis. Given these findings, it was decided to perform an endovascular treatment. Significant stenosis was seen during the arteriography in both renal arteries, suggesting fibromuscular dysplasia and development of a collateral neovascular network responsible for the retroperitoneal haematoma. It was embolised in association with angioplasty of the left renal artery. The patient had a favourable outcome; however, high blood pressure levels persisted. A new bilateral renal angioplasty was performed, which returned blood pressure values to normal. The patient was discharged without needing antihypertensives.

**Conclusions:**

FMD is a rare disease that can show multiple clinical presentations and need individualized treatment options. Endovascular techniques are in the first therapeutic line regarding fibromuscular dysplasia.

## Background

Fibromuscular dysplasia (FMD) is an uncommon vascular disease that results in stenosis (either focal or more frequently multiple in string of beads), dissection or aneurysmal degeneration of medium-sized arteries, characteristically renal arteries and extracranial carotids. FMD results from the abnormal development of the arterial wall, most commonly of the medial, though the intima can be also affected (Doody et al., 2009). Its cause is not completely known, however it could be secondary to a combination of genetic, hormonal and/or environmental factors (Doody et al., 2009; Brinza & Gornik, 2016). It generally affects young or middle-aged women and clinically occurs as headache, hypertensive crises or tinnitus (Doody et al., 2009; Brinza & Gornik, 2016). Unlike atherosclerotic conditions, FMD is not associated with typical cardiovascular risk factors, except for smoking (Brinza & Gornik, 2016).

Endovascular techniques play an important role concerning the FMD treatment.

## Case presentation

**Fig. 1:**
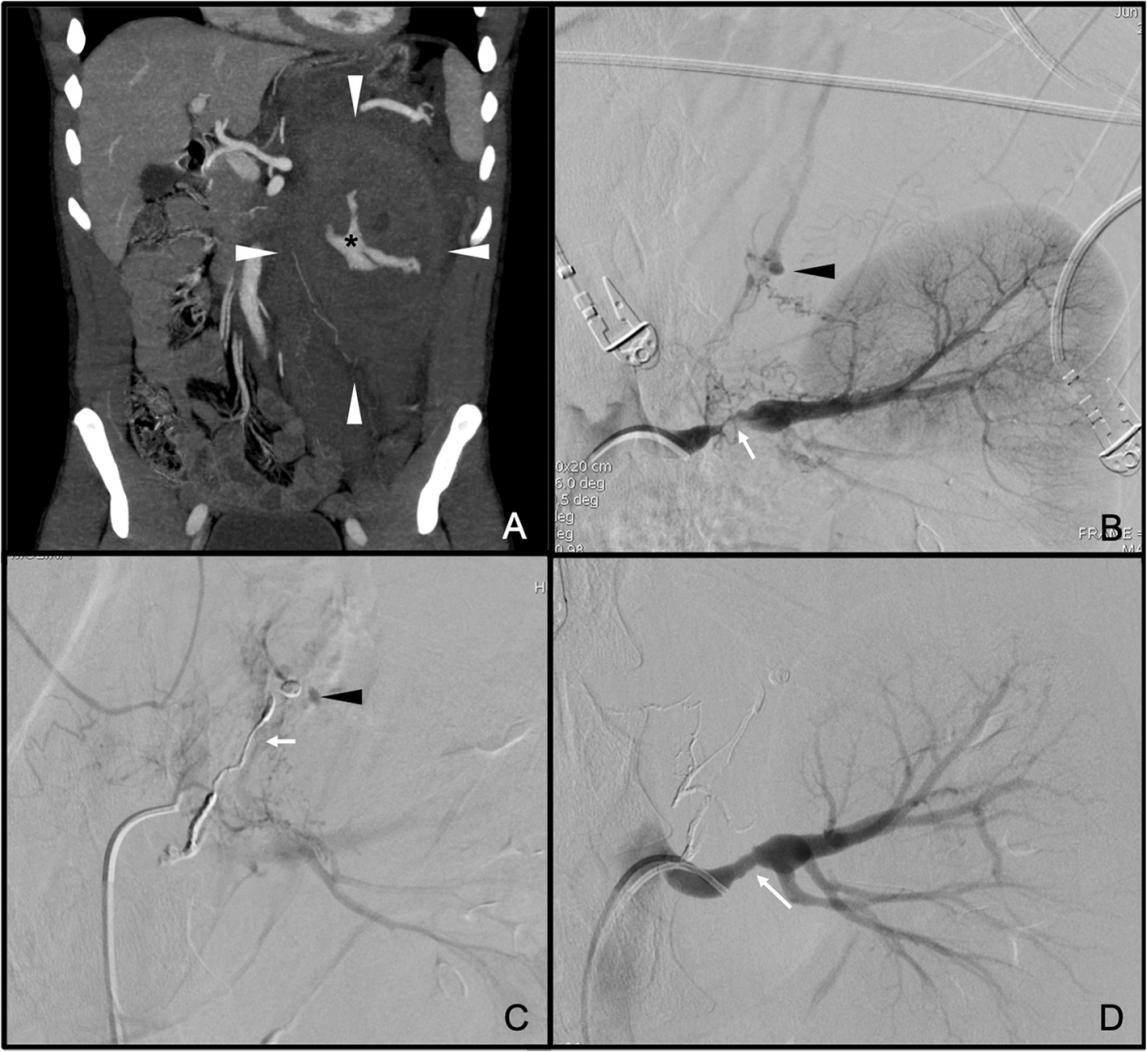
**a** Angio-CT, coronal plane. Left retroperitoneal haematoma (white arrowheads), with active bleeding inside (black asterisk). **b** Selective arteriography of the left renal artery, with contrast extravasation (black arrowhead) dependent on vascular network, and stenosis of the renal artery (white arrow). **c** Embolisation material in the vascular network (white arrow), new contrast extravasation point dependent on adrenal aortic branch (black arrowhead). **d** Control after embolisation and angioplasty, without identifying contrast extravasation points and with partial recovery of the stenosis (white arrow)

We report the case of a 24-year old male patient, with unrelevant past medical history, who went to the emergency department due to sudden, non-traumatic, left hypochondrium severe pain. The patient reported to suffer frequent headaches that started 2 years before and subsided taking NSAIDs. On the physical examination the patient was eupnoeic and haemodynamically stable, though, despite being considered normotensive (reporting normal blood pressure levels in several previous medical examinations), he had blood pressure levels of 160–90 mmHg on arrival, which increased to 180 mmHg of systolic blood pressure. The laboratory tests performed did not show remarkable disorders. An abdominopelvic CT was performed with intravenous contrast (iv), which evidenced the presence of a large retroperitoneal haematoma of 24 × 9 × 8.5 cm in diameters and active extravasation of contrast in the left adrenal area, but the responsible vessel could not be established. Stenosis of the left renal artery and free peritoneal fluid were also seen (Fig. 1a).

An urgent arteriography was performed, viewing significant bilateral renal stenosis. After the selective angiographic series of the left renal artery, the existence of a profuse collateral vascular network to the adrenal gland with signs of active bleeding was seen (Fig. 1b). This network could be embolised completely by injecting Onyx (Medtronic ev3 Neurovascular Irvine CA. USA). After this, another arterial branch, which also nourished another part of this network, evidencing signs of active bleeding was detected (Fig. 1c). It was embolised in the same way by injecting Onyx. Finally, after an extensive vascular study confirming no other sign of active bleeding, an angioplasty of the left renal artery was performed using a Mustang balloon catheter (Boston Scientific Corporation. Malborough MA. USA) of 4 mm × 4 cm, with moderate recovery of calibre (Fig. 1d).

**Fig. 2:**
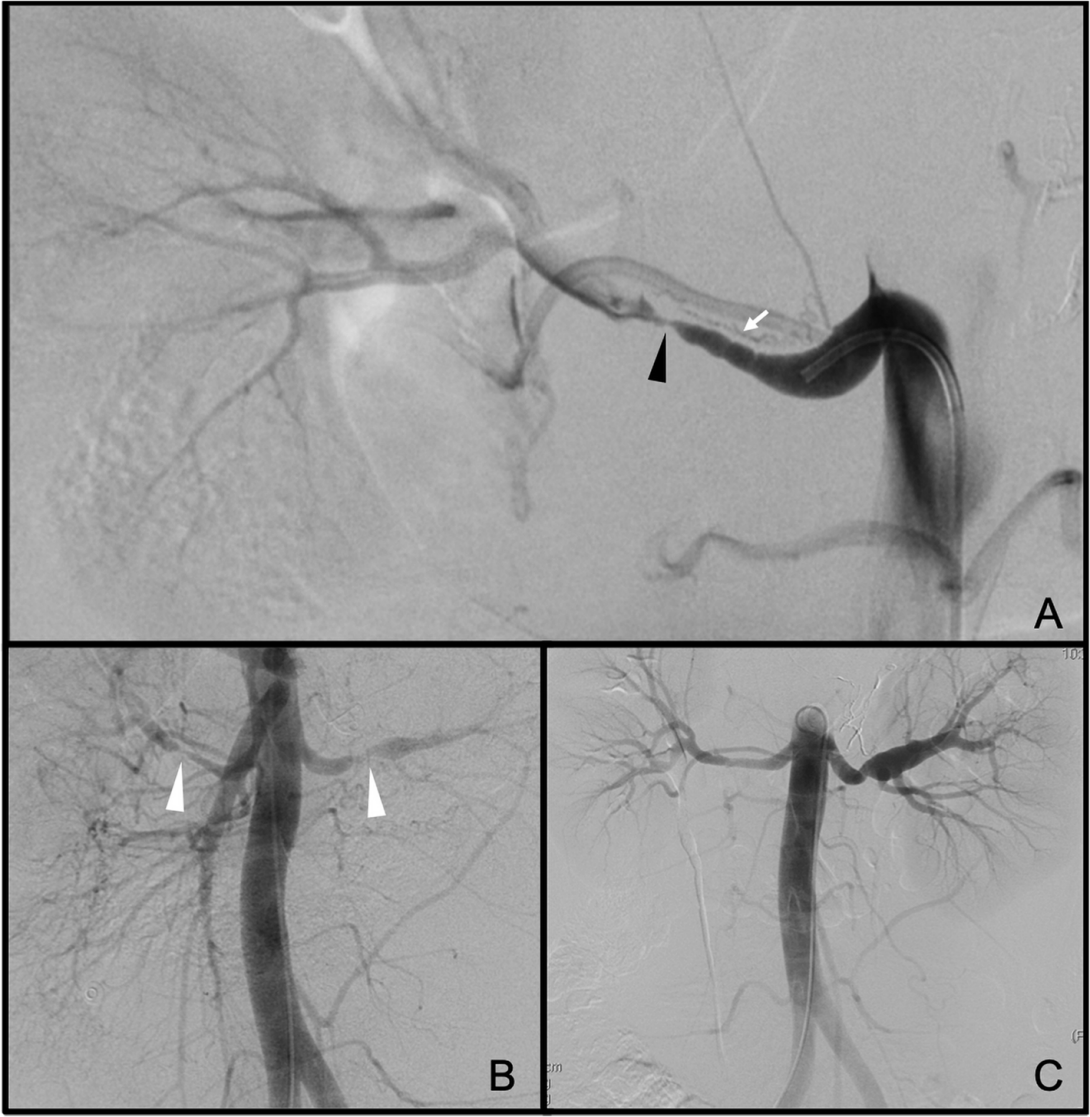
**a** Stenosis of the right renal artery (black arrowhead) with string of beads appearance (white arrow). **b** Pre-treatment aortogram with stenosis of both renal arteries (white arrowhead). **c** Aortogram after bilateral angioplasty, with resolution of the stenosis

The patient had a favourable outcome, though he had a fever peak of 37.9 °C (with negative urine and blood cultures), so levofloxacin antibiotic therapy was instituted for preventive purposes. He had very high blood pressure levels, with peaks up to 200 mmHg, requiring administration of Amlodipine, Doxazosin and even intravenous Urapidil pulses for controlling them. Given this situation, it was decided to perform a new renal angioplasty, in this case bilateral, using a Mustang balloon catheter of 6 mm × 4 cm (Boston Scientific Corporation. Malborough MA. USA) achieving full bilateral recovery of the normal renal artery calibre (Fig. 2). After this, the patient’s blood pressure levels returned to normal.

During his hospital stay, he underwent Doppler ultrasound of the supra-aortic trunks and transthoracic echocardiogram showing no pathological disorders.

The patient was discharged at 16 days of admission, asymptomatic, fever-free, not requiring medical treatment and with the only recommendations of avoiding intense exercise and a salt-free diet.

Due to the hospital situation caused by COVID-19, all face-to-face patient review consultations were suspended in our centre. Recently, we were able to carry out an evolutionary assessment of the patient by phone (eleven months after the endovascular treatment) in which he declared to be asymptomatic, maintaining normal blood pressure without the need for medical treatment.

## Discussion

Retroperitoneal haematoma is a relatively rare pathological condition and most cases are related with ruptured aortic or iliac aneurysm, or lumbar trauma. Patients may present with back or lower abdominal pain, hypotension, haemodynamic instability and decreased haemoglobin levels (Chan et al., 2008). Urgent CT with i.v. is mandatory in order to assess haematoma localization and extent, and also to identify active bleeding and possible underlying causes. In patients with hemodynamic instability or confirmed active bleeding urgent angiography is indicated with intention to embolize. The endovascular embolization can be performed with multiple embolic agents including coils, particles, liquid agents as N-butyl cyanoacrylate glue, Onyx, absorbable gelatin sponge and others. There is no consensus on which embolic agent to use, so the decision must be based on the differential characteristics of each case and the operator’s experience with each agent. Onyx, despite its use is technically demanding, was the chosen option for the case previously presented above because it is effective regardless any underlying coagulopathy, and its differential characteristics (permanent embolic agent, no need of free flow to embolize, minimal risk of reflux, no risk of microcatheter entrapment) were considered advantageous in embolizing the vascular network responsible for the bleeding. There is wide evidence in the literature that Onyx is safe and effective in the treatment of retroperitoneal haemorrhage (Mahdjoub et al., 2020; Kolber et al., 2015), and the final result was excellent, achieving a complete embolization as detailed in the case presentation. The major complication rate as Kolber et al. (Kolber et al., 2015) point out in a systematic review article is under 5%.

The association of FMD with a retroperitoneal haematoma is very infrequent, although it has been previously described in the literature. In most cases it is related to the presence of a mass, or a vascular rupture from an aneurysm or a dissection (Phillips & Lepor, 2006; Shimada et al., 2009). However none of these conditions was identified in our case. The authors think that the bleeding was probably the product of the rupture of the collateral network as a consequence of a hypertensive crisis. The development of collaterals in the context of FMD is not common, but its presence in the context of FMD has been previously described (Sekar & Shankar, 2013).

Once the embolization was performed and the bleeding was controlled, the operator focused on the detailed study of FMD vascular lesions and its need for urgent treatment. It was decided to perform urgent angioplasty of the left renal artery given its intense degree of stenosis, considering performing the treatment of the right one in a second stage.

FMD comprises a group of pathological conditions of unknown aetiology, that cause stenosis of non-atheromatous origin in arteries of medium or small calibre (Doody et al., 2009; Brinza & Gornik, 2016). Due to the fact that it is an infrequent pathology, the majority of the scientific evidence is derived from descriptive articles and systematic reviews. The association of fibrous proliferation foci and segments with collagen loss causes the typical occurrence in string of beads, alternating segmental stenosis (fibrotic foci) with pseudodilatations (collagen loss). It generally affects Caucasian women aged 15–50 years. The main target are renal and extracranial carotid arteries, though it has been described in multiple arterial locations (Brinza & Gornik, 2016). Symptoms are variable based on the location and severity of the involvement. The most common are hypertension, headache and pulsatile tinnitus and cervical pain or dizziness. Other symptoms can also occur, such as stroke, renal fossa pain, myocardial infarction (Doody et al., 2009; Brinza & Gornik, 2016), and even retroperitoneal haematoma (Phillips & Lepor, 2006; Shimada et al., 2009).

In most cases non-invasive imaging studies (Angio-CT, Angio-MRI and Doppler) are enough to make the diagnosis. Arteriography is reserved for cases in which there are diagnostic doubts or for endovascular treatment (Gottsäter & Lindblad, 2014).

There is no established treatment regimen for FMD since the treatment is usually individualised (Doody et al., 2009; Brinza & Gornik, 2016). Background treatment with platelet aggregation inhibitors or ACEIs is frequent (Brinza & Gornik, 2016). Considering endovascular treatment renal angioplasty (RA) has become the treatment of choice. RA can be performed not only for the main renal artery but also when branch arteries are affected (Gottsäter & Lindblad, 2014). Balloon-catheter size is recommended to be 10–20% greater than the expected artery diameter but sometimes it can be difficult to estimate due to extensive disease, in those cases it is best to underestimate balloon size and redilate with a larger balloon if needed (Meuse et al., 2010). Technical success rates are around 100% (Mousa & Gill, 2013) and patency rates following RA have been reported from 80 to 90% over 10 years (Meuse et al., 2010), however they may vary depending on the type of FMD affectation, reporting worse patency rates in multifocal FMD compared to unifocal FMD (Gottsäter & Lindblad, 2014). Major complications after RA have been reported below 10% (Barrier et al., 2010). Cutting-balloon angioplasty is usually reserved to treat lesions resistant to conventional angioplasty. Typically cutting-balloons are undersized to the target artery diameter by 1 mm to decrease the risk of vessel rupture, which is a well known complication. Stents and stent-grafts are reserved almost exclusively for treating angioplasty complications such as a dissection or vessel rupture (Meuse et al., 2010). Other techniques like renal artery denervation therapy or drug coated-balloon angioplasty has been recently described obtaining good outcomes (Mousa & Gill, 2013; Kelle et al., 2013; Morosetti et al., 2018) however more studies are needed before generalizing these new therapy options in the context of FMD. Surgical therapy is currently reserved for patients with patients with severe complications that cannot be handle with endovascular techniques (Gottsäter & Lindblad, 2014). Finally, recommendations such as quitting smoking, keeping an adequate weight, avoiding extreme exercise or contact sports, are also very common and highly important.

## Conclusion

FMD is an uncommon vascular disease that rarely manifests clinically as a retroperitoneal haematoma. This report highlights that endovascular procedures are safe, feasible and technically successful in treating both conditions and should be considered as a first line treatment.

## Data Availability

Data sharing is not applicable to this article as no datasets were generated or analysed during the current study.

## Abbreviations

FMD: Fibromuscular dysplasia
iv: Intravenous contrast
RA: Renal angioplasty
